# Case Report: Endogenous endophthalmitis caused by *Listeria monocytogenes* infection in patients with end-stage renal disease

**DOI:** 10.3389/fmed.2025.1643137

**Published:** 2025-07-24

**Authors:** Kaichuan Chen, Xiao Lyu, Xin Liu, Yanlong Bi, Zhen Wang

**Affiliations:** ^1^Department of Ophthalmology, Baoshan Branch, Ren Ji Hospital, School of Medicine, Shanghai Jiao Tong University, Shanghai, China; ^2^Department of Ophthalmology, Tongji Hospital, School of Medicine, Tongji University, Shanghai, China

**Keywords:** *Listeria monocytogenes*, endogenous endophthalmitis, pathogenic microbial metagenome, end-stage renal disease, intraocular aqueous

## Abstract

**Purpose:**

This paper reports a rare case of endogenous *Listeria monocytogenes* endophthalmitis caused by hemodialysis in end-stage renal disease (ESRD).

**Methods:**

The patient was a 70-year-old female. The best-corrected visual acuity (BCVA) was light perception (LP). Endogenous endophthalmitis was diagnosed based on clinical manifestations and auxiliary examinations. Pathogenic microbial metagenomic detection of intraocular aqueous was used to identify *Listeria monocytogenes*. As multiple investigations showed no source of infection, a standardized treatment plan was proposed.

**Results:**

The treatment included the local application of antibiotics, glucocorticoids and cycloplegic drugs, vitrectomy, intravitreal injection of vancomycin and ceftazidime, anterior chamber irrigation, and systemic antibiotic treatment after the operation. After treatment, the inflammation subsided, and the BCVA at discharge was hand movement (HM).

**Conclusion:**

Our study highlights the importance of rapid pathogenic microbial metagenomic detection and timely standardized intervention in the treatment of endogenous endophthalmitis.

## Introduction

Endophthalmitis is mostly caused by exogenous pathogens, with endogenous pathogens accounting for only 5% to 10% of cases (1). *Listeria monocytogenes* is a rare and serious pathogen (1). Listeria is usually acquired by eating contaminated food, especially for susceptible populations such as pregnant women, newborns, the elderly and immunocompromised people. Infection can lead to serious diseases such as meningitis and sepsis. Since symptoms may appear in a few days to a few weeks, the link to contaminated food is usually not noticed (2). Although the global incidence of listeriosis is relatively low, ranging from 0.1 to 10 cases per million people per year, the hospitalization rate is more than 90%, and the mortality rate is 20% to 50%. This bacterial pathogen poses a major threat to human health. Therefore, antibiotic treatment is usually required to control *Listeria monocytogenes* infection (3). *Listeria* endogenous endophthalmitis has been reported in patients with both normal and compromised immune functions (4).

Pathogenic microbial metagenome is a new technology for the diagnosis of pathogenic microorganisms in intraocular aqueous characterized by its rapidity and efficiency. We report a case of endogenous endophthalmitis caused by *Listeria monocytogenes* in a patient with end-stage renal disease (ESRD).

## Case report

**Figure 1:**
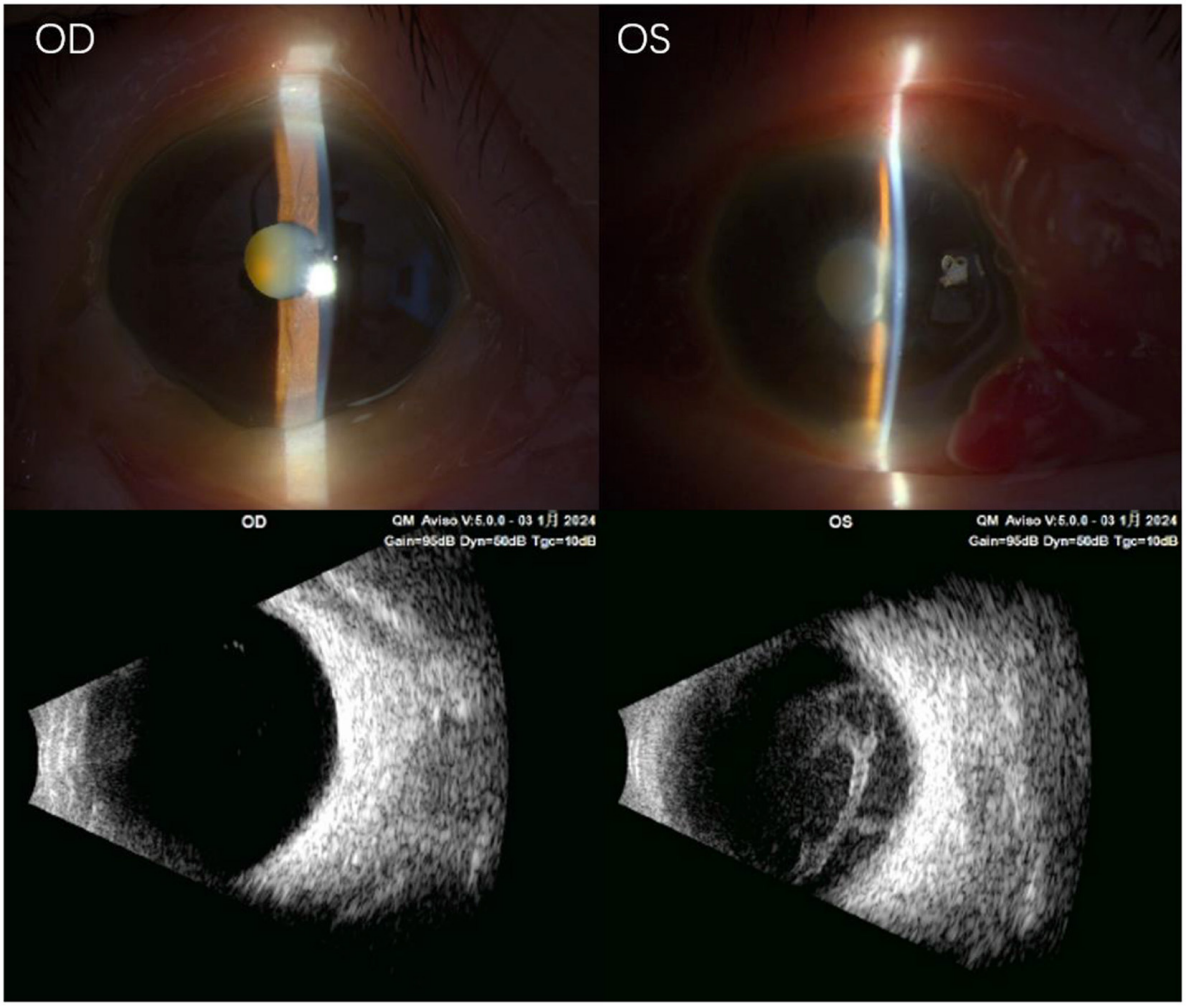
Preoperative auxiliary examination. The images from the slit lamp microscope showed no obvious abnormalities in the anterior chamber of the right eye, although the lens was turbid. However, the left eye showed conjunctival congestion, severe edema of the temporal conjunctiva, mild corneal edema, and fibrinous exudation in the anterior chamber, with pus under the anterior chamber. Ultrasound examination of the right eye showed a small amount of punctate opacity in the vitreous cavity, while the left eye had vitreous mass opacity and vitreous infiltration with retinal edema. OD, right eye; OS, left eye.

A 70-year-old female was referred to the Department of Ophthalmology by a nephrologist due to sudden vision loss in her left eye for 4 days. The patient's left eye examination showed that the best-corrected visual acuity (BCVA) was light perception (LP). Conjunctiva showed diffuse congestion, corneal transparency remained, no obvious edema or infiltration. The anterior chamber depth was normal, but fibrinous exudation with pus formation could be seen. The pupil diameter was about 5.0 mm, showing a moderate degree of astigmatism. The direct and indirect light reflection was slow, and the accommodative reflection disappeared. The lens showed grade 3 nuclear opacity. There is dense foggy opacity in the vitreous cavity, and the fundus details cannot be seen (Figure 1). A B-ultrasound examination of the posterior segment of the eye revealed vitreous inflammation (Figure 1). Intraocular pressure was normal. The patient's general health status was fair, with no history of eye surgery or trauma. She had a 2-year history of hypertension, treated with nifedipine and sacubitril, and well-controlled blood pressure. She had previously been diagnosed with ESRD, and the etiology of ESRD was unknown. Recent laboratory blood tests found glomerular filtration rate (GFR) 9.0 mL/(min·1.73m^2^), creatinine (Cr) 444.1 μmol/L, urea 26.1 mmol/L, hemoglobin (HB) 79 g/L, red blood cell (RBC) 2.74 × 10 ∧ 12/L, platelet (PLT) 114 × 10 ∧ 9/L, hematocrit (HCT) 27.3 %, neutrophil ratio 79.1 %, total protein (TP) 52.7 g/L, albumin (ALB) 26.4 g/L. She began hemodialysis in May 2022, twice a week, with a long-term catheter in place and heparin anticoagulant therapy. No recent infection or travel was reported. Therefore, the initial diagnosis is endophthalmitis of the left eye, chronic hypertension, ESRD, and renal anemia.

**Figure 2:**
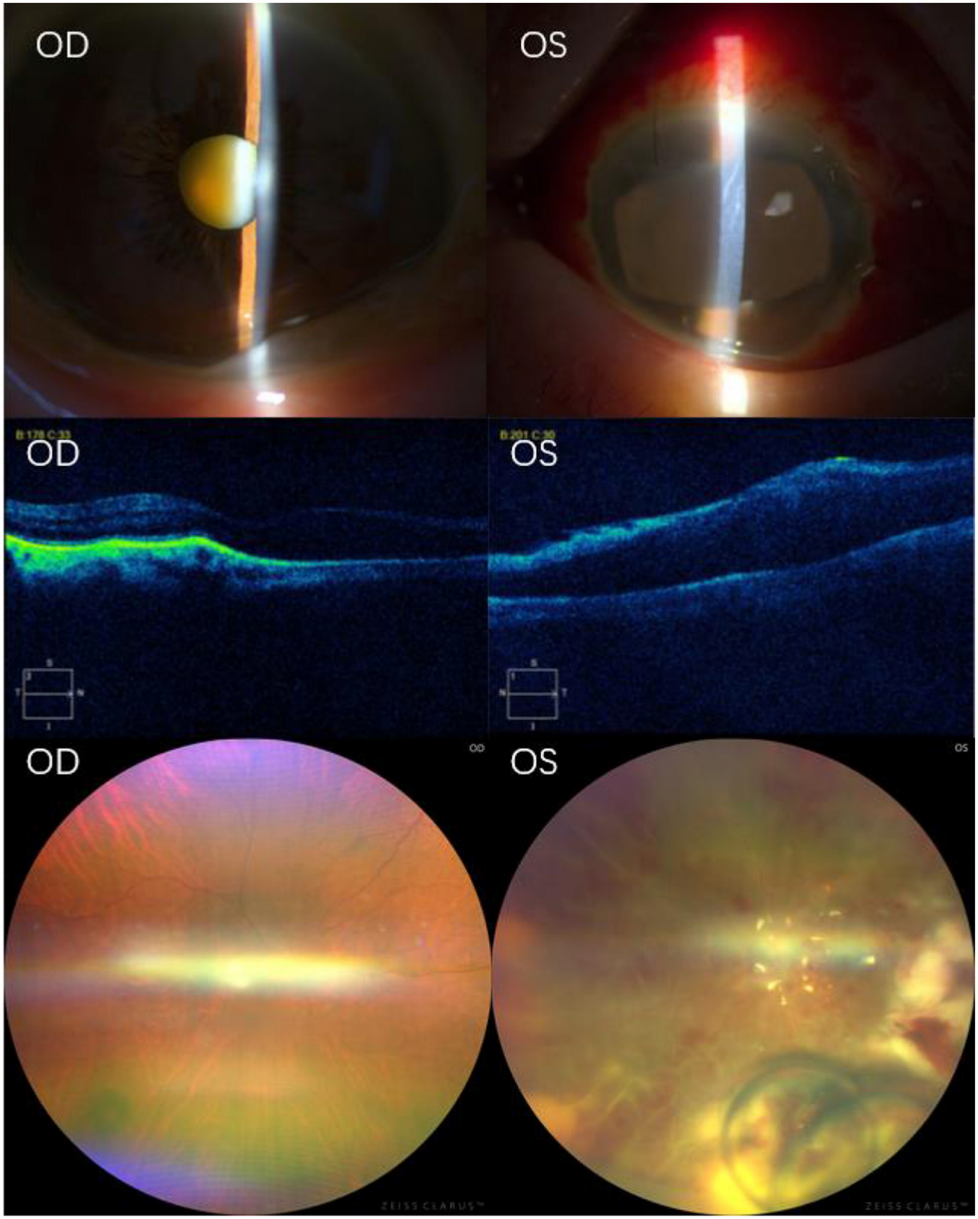
Postoperative auxiliary examination. Slit-lamp microscopy showed no obvious abnormalities in the anterior chamber of the right eye, with the lens still turbid. The left eye exhibited mixed conjunctival hyperemia, mild corneal edema, folds in the corneal Descemet's membrane, a small amount of inflammatory cell exudation in the anterior chamber, irregularly dilated pupil with a sluggish light reflex, and lens removal. OCT examination showed that the retinal pigment epithelium of the right eye was slightly elevated, with no abnormalities in the neuroepithelial layer. However, in the left eye, the retina in the macular area was slightly elevated, and the rest of the structure was blurred. Fundus photography showed no obvious abnormalities in the retina of the right eye. The left eye exhibited extensive retinal edema, hemorrhage, white linear occlusion of blood vessels, and white necrotic lesions of the retina on the nasal and lower periphery. Vancomycin and ceftazidime liquid droplets were observed below. OD, right eye; OS, left eye; OCT, optical coherence tomography.

Initial treatment included immediate intravenous infusion of vancomycin and ceftazidime and topical administration of tobramycin dexamethasone eye drops, levofloxacin eye drops, ofloxacin eye ointment, and tropicamide eye drops as adjuvant therapy. The patient was admitted to the Department of Ophthalmology for monitoring and general examination. Blood analysis showed a slight increase in the erythrocyte sedimentation rate and C-reactive protein. Considering the emergency of the disease, the next day, under local anesthesia, left eye phacoemulsification, vitreous diagnostic aspiration, and posterior vitrectomy were performed. Vitreous and aqueous humor samples were obtained for pathogenic microbial metagenomic detection. At the same time, vancomycin and ceftazidime were injected into the vitreous cavity of the left eye, and finally, silicone oil was implanted into the vitreous cavity. Three days later, the results of the aqueous and vitreous humor showed the growth of Gram-positive bacteria, which were identified as *Listeria monocytogenes*. Further investigation of the cause of infection (blood culture, abdominal ultrasound, and chest X-ray) was carried out during hospitalization. However, no primary infection was found. The final diagnosis was endogenous *Listeria monocytogenes* endophthalmitis in the left eye. After the operation, the patient was treated with an intravenous infusion of vancomycin and cefuroxime sodium (once daily) and the local administration of tobramycin dexamethasone eye drops (four times daily), levofloxacin eye drops (four times daily), tropicamide eye drops (three times daily), and ofloxacin eye ointment (once daily) as adjuvant therapy. After treatment, the BCVA of the left eye was hand movement (HM), and the intraocular pressure was 10 mmHg. The patient exhibited conjunctival mixed congestion, a clear cornea, a clear anterior chamber, mydriasis, and aphakia, and the fundus was filled with silicone oil, with the retina flat (Figure 2). After the patient's ophthalmic condition stabilized, the systemic antibiotic treatment was discontinued, and she was transferred to the Department of Nephrology.

## Discussion

*Listeria monocytogenes* is a Gram-positive bacterium that can cause invasive syndromes. It has been reported in the literature that the pathogen causes endophthalmitis, keratitis, corneal ulcers, and conjunctivitis. Infection pathways include blood-borne and exogenous infections caused by contact with pollutants (5). Endogenous endophthalmitis is caused by the hematogenous spread of microorganisms through the blood barrier into the eyes. It is estimated that *Listeria monocytogenes* accounts for 4% of endogenous causes (6). This report describes a rare case of endogenous endophthalmitis caused by *Listeria monocytogenes* bacteremia, which may be associated with ESRD. ESRD is a known state of immune damage that makes patients susceptible to infection. Diseases caused by *Listeria monocytogenes* can affect any host but are often more severe in immunocompromised patients (7). In this report, the patient did not exhibit obvious primary infection lesions but had a history of ESRD and underwent hemodialysis twice a week from May 2022. Although there is no evidence that hemodialysis is significantly associated with *Listeria monocytogenes* infection, previous studies have shown that this type of infection is common in patients with cirrhosis and those undergoing peritoneal dialysis, spreading from lymph nodes to other organs through blood flow (8). Considering that the patient was diagnosed with ESRD and had low immune function, we highly suspect that hemodialysis created conditions conducive to the invasion of *Listeria monocytogenes*.

The diagnosis of endogenous *Listeria monocytogenes* endophthalmitis is rare and usually unexpected, especially in patients with asymptomatic bacteremia and no further history, often leading to delayed diagnosis (9). The typical clinical manifestations of *Listeria monocytogenes* endophthalmitis include a cellulose anterior chamber reaction with elevated intraocular pressure, and varying degrees of vitreous and retinal involvement. Although pigmented anterior chamber empyema has occasionally been observed, no characteristic sign of *Listeria monocytogenes* has been reported.^(6)^ Therefore, in cases of low paracentesis, especially dark low paracentesis with high intraocular pressure, *Listeria* infection should be considered. The presence of *Listeria monocytogenes* can be confirmed by Gram staining, PCR, or culture (10); however, these detection methods take 5–7 days.

As a new detection technology, microbial metagenome does not depend on the isolation and culture of pathogens. It can obtain all microbial DNA information from a small number of specimens, and can obtain low abundance pathogen DNA in the environment, and even capture residual pathogen biological information. The metagenome sequencing turnaround time varies according to the platform, and the average time of detection results is only 24 h (11). Relying on metagenomic sequencing technology, pathogen identification and quantitative detection can be carried out quickly. This method can accurately and efficiently assist in the diagnosis of diseases and provide guidance for doctors to formulate treatment plans. In this report, we used the pathogenic microbial metagenome to analyze aqueous and vitreous humor samples obtained during the operation, confirming the presence of *Listeria monocytogenes*, a procedure that took only 3 days. This not only proves that the pathogenic microbial metagenome is rapid and efficient but also emphasizes the equal importance of aqueous and vitreous humor in the diagnosis of endogenous endophthalmitis.

At present, there is no standard treatment for *Listeria monocytogenes*. As with all cases of endophthalmitis, timely intravitreal injection of antibiotics is a key factor in improving vision (12). A recent review article showed that *Listeria monocytogenes* was sensitive to vancomycin, and most strains were resistant to ceftazidime (13). Chersich et al. (2) found that many drugs for the treatment of *Listeria monocytogenes* have poor penetration of the eye and have different penetrations in the anterior and posterior segments of the eye. Ampicillin or penicillin G is the preferred antibiotic for the treatment of *Listeria monocytogenes*. Unfortunately, the patient had an allergic reaction to penicillin G, but the study showed (2) that no live bacilli were isolated from the same eye specimens after treatment with cephalosporins. Therefore, different treatments are used in different cases, which indicates that there is a clear uncertainty about the optimal treatment for this rare disease. We injected vancomycin and ceftazidime into the vitreous cavity of the patient during the operation to control further inflammation. Given that the most common infection route of *Listeria monocytogenes* endophthalmitis is endogenous, systemic antibiotic therapy is also recommended. Therefore, the patient was given systemic antibiotic therapy before and after the operation. When endophthalmitis occurs, the damaged blood–retinal barrier is more permeable, allowing systemic antibiotics to reach intraocular therapeutic concentrations (14). The final treatment effect mainly depends on the degree of retinal involvement. The majority of patients with endogenous *Listeria monocytogenes* endophthalmitis have poor vision even after appropriate antibiotic treatment, as this rare pathology can easily lead to delayed diagnosis (15). In this report, the inflammatory reaction in the patient's left eye subsided after treatment, and the BCVA of the left eye improved from LP to HM. The B-mode ultrasound scan of the eye showed that the silicone oil filled the left eye, the retina was annularly attached, and there was no opacity in the vitreous cavity.

In conclusion, endogenous *Listeria monocytogenes* endophthalmitis is rare but can lead to poor visual prognosis and even severe systemic diseases. In the treatment of patients with a large number of fibrous exudates with anterior chamber abscesses, potential differential diagnoses should be considered. *Listeria monocytogenes* endophthalmitis can only be diagnosed after microbial detection, which may delay the timely use of antibiotics. Timely detection of pathogenic microbial metagenome in intraocular aqueous and early standardized treatment are very important for the prognosis of patients with endogenous *Listeria monocytogenes* endophthalmitis.

## Data Availability

The original contributions presented in the study are included in the article/supplementary material, further inquiries can be directed to the corresponding author.
